# Phylogenetic and morphometric analyses reveal ecophenotypic plasticity in freshwater mussels *Obovaria jacksoniana* and *Villosa arkansasensis* (Bivalvia: Unionidae)

**DOI:** 10.1002/ece3.649

**Published:** 2013-07-03

**Authors:** Kentaro Inoue, David M Hayes, John L Harris, Alan D Christian

**Affiliations:** 1 Environmental Sciences Graduate Program, Arkansas State UniversityP.O. Box 877, State University, Arkansas, 72467; 2 Department of Zoology, Miami University700 High Street, 212 Pearson Hall, Oxford, Ohio, 45056; 3 Department of Biological Sciences, Eastern Kentucky University521 Lancaster Avenue, 235 Moore Building, Richmond, Kentucky, 40475; 4 Department of Biological Sciences, Arkansas State UniversityP.O Box 599, State University, Arkansas, 72467; 5 Biology Department, University of Massachusetts at Boston100 Morrissey Boulevard, Boston, Massachusetts, 02125

**Keywords:** Isolation by distance, Law of Stream Distribution, Mantel test, mitochondrial DNA, Ortmann's law, phenotypic plasticity

## Abstract

Freshwater mollusk shell morphology exhibits clinal variation along a stream continuum that has been termed the Law of Stream Distribution. We analyzed phylogenetic relationships and morphological similarity of two freshwater mussels (Bivalvia: Unionidae), *Obovaria jacksoniana* and *Villosa arkansasensis*, throughout their ranges. The objectives were to investigate phylogenetic structure and evolutionary divergence of *O. jacksoniana* and *V. arkansasensis* and morphological similarity between the two species. Our analyses were the first explicit tests of phenotypic plasticity in shell morphologies using a combination of genetics and morphometrics. We conducted phylogenetic analyses of mitochondrial DNA (1416 bp; two genes) and morphometric analyses for 135 individuals of *O. jacksoniana* and *V. arkansasensis* from 12 streams. We examined correlations among genetic, morphological, and spatial distances using Mantel tests. Molecular phylogenetic analyses revealed a monophyletic relationship between *O. jacksoniana* and *V. arkansasensis*. Within this *O. jacksoniana/V. arkansasensis* complex, five distinct clades corresponding to drainage patterns showed high genetic divergence. Morphometric analysis revealed relative differences in shell morphologies between the two currently recognized species. We conclude that morphological differences between the two species are caused by ecophenotypic plasticity. A series of Mantel tests showed regional and local genetic isolation by distance. We observed clear positive correlations between morphological and geographic distances within a single drainage. We did not observe correlations between genetic and morphological distances. Phylogenetic analyses suggest *O. jacksoniana* and *V. arkansasensis* are synonomous and most closely related to a clade composed of *O*. *retusa*,* O*. *subrotunda*, and *O*. *unicolor*. Therefore, the synonomous *O. jacksoniana* and *V. arkansasensis* should be recognized as *Obovaria arkansasensis* (Lea 1862) n. comb. Phylogenetic analyses also showed relative genetic isolation among drainages, suggesting no current gene flow. Further investigation of in-progress speciation and/or cryptic species within *O. arkansasensis* is warranted followed by appropriate revision of conservation management designations.

In this study, we found *Obovaria jacksoniana* and *Villosa arkansasensis* are synonomous. We suggest that morphological differences between the two species are caused by ecophenotypic plasticity, where *V. arkansasensis* is the upstream morphotype and *O. jacksoniana* is the downstream morphotype of a single species.

## Introduction

Preservation of biological diversity is the fundamental goal of conservation biology; however, the total biodiversity on Earth is still undetermined. Although freshwater ecosystems represent a small portion of the global area, they harbor a disproportionally high level of diversity (Dudgeon et al. 18; Strayer and Dudgeon 70). Meanwhile, freshwater ecosystems may be the most threatened due to human use and associated activities causing worldwide habitat degradation. Examples of habitat degradation include a decline in water quality by pollution, over exploitation of biota, introduction of invasive species, and modification of natural flow (Dudgeon et al. 18). These activities caused severe declines in the habitat quality, range, and abundance of many freshwater species. In fact, the biodiversity extinction rate for freshwater ecosystems is increasing at a much faster rate than for terrestrial ecosystems (Ricciardi and Rasmussen 59). Even limited data on extinction rates in North America freshwater ecosystems are indicative of a global freshwater biodiversity crisis (Abell et al. 1).

Freshwater mussels (Bivalvia: Unionoida) are among the most endangered groups of animals in North America (Williams et al. 81; Neves 51; Lydeard et al. 43; Strayer et al. 71; Haag 31). Of the approximately 300 recognized freshwater mussel species in North America, approximately 72% are endangered, threatened, proposed for listing or presumed extinct (Williams et al. 81; Lydeard et al. 43). Conservation efforts are hindered by taxonomic uncertainty and a lack of ecological and evolutionary information on most taxa (Strayer et al. 71).

Accurate species identification is necessary to assess management options for freshwater mussel conservation and protection. External morphology is the primary characteristic for freshwater mussel species identification; however, morphology-based identification is problematic and may lead to misidentifications due to shell erosion and cryptic morphology. Furthermore, shell morphologies are highly variable due to environmental factors, sometimes resulting in extreme ecophenotypic plasticity within single species (Watters 80). In other organisms, morphology often exhibits trends of clinal variation within single species reflecting positional (e.g., latitude, longitude, altitude, and depth) and/or environmental (e.g., temperature, precipitation, and salinity) variables (Gaston et al. 25). Morphological variations in freshwater mussels are thought to be caused by a combination of hydrodynamic forces, substrate types, burrowing activities, and/or historic environmental conditions (Watters 80; Peacock and Seltzer 55; Allen and Vaughn 2). Several studies have observed morphological variations of the shell among populations in different habitats (Utterback 76; Ortmann 54; Ball 4; Mackie and Topping 44; Hornbach et al. 35; see Haag 31 for a summary). An early study reported that a single species could be light and laterally compressed in form in headwaters, but heavier and more laterally inflated in downstream waters (Utterback 76). Detailed observations on the upper Tennessee River fauna reported differences in shell inflation correlating with river positions (Ortmann 53). Ortmann (54) termed this clinal morphological variation the Law of Stream Distribution, where gradual changes in shell morphologies occur from upstream to downstream. Although several studies have observed phenotypic plasticity in shell morphologies, to date few have explicitly tested the Law of Stream Distribution in freshwater mollusks (e.g., Mackie and Topping 44; Minton et al. 48, 49).

**Figure 1:**
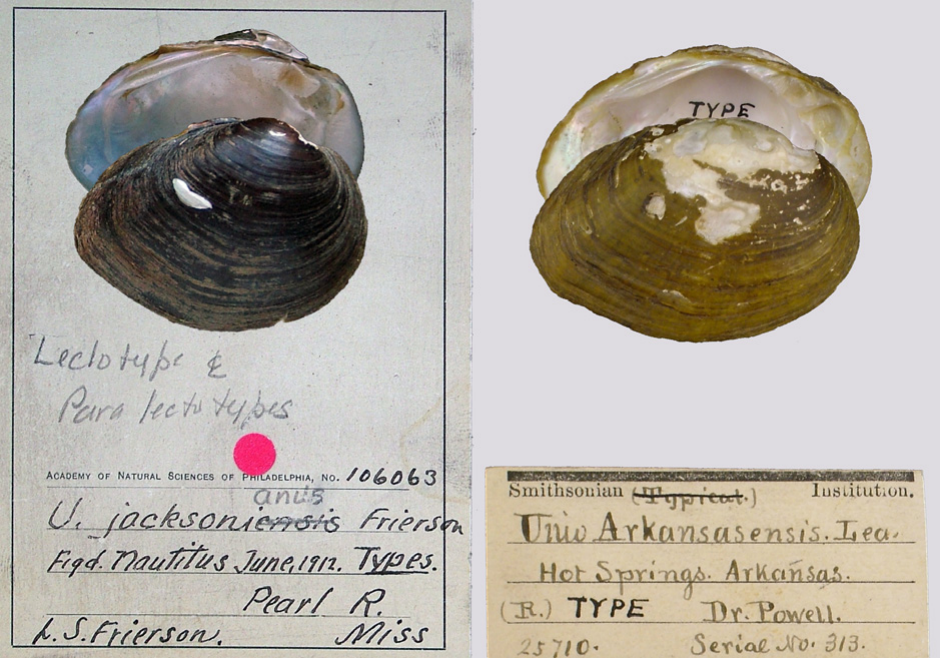
Type specimens of *Obovaria jacksoniana* (left; Academy of Natural Sciences of Drexel University, ANSP 106063) and *Villosa arkansasensis* (right; Smithsonian National Museum of Natural History, USNM 25710). The pictures were provided by the MUSSEL project (http://mussel-project.uwsp.edu).

We used molecular phylogenetics and shell morphometrics to investigate the relationship between *Obovaria jacksoniana* (Frierson 23) and *Villosa arkansasensis* (Lea 42) (Fig. 1). These two freshwater mussel species are sometimes confused (Vaughn et al. 77) and are of conservation concern (Williams et al. 81; Harris et al. 33). *O. jacksoniana* is widely distributed from the Interior Highlands to the Gulf of Mexico and Mobile River basins, and inhabits mid- to large-size rivers (Oesch 52; Howells et al. 36). Although it is locally abundant in preferred habitat, this species is globally rare (Branson 7; Vaughn et al. 77; NatureServe 50). The *O. jacksoniana* type locality is the Pearl and Yalobusha rivers in Mississippi (Frierson 23; Fig. 1). A recent survey of the lower Pearl River did find any live specimens (Brown et al. 9). *V. arkansasensis* is endemic to the Ouachita Mountains region in Arkansas and Oklahoma, and inhabits headwaters to small-size rivers in the Ouachita and Red River systems (Robison and Allen 60). The *V. arkansasensis* type locality is the Ouachita River near Hot Springs, Arkansas (Lea 42; Fig. 1); however, it is presumably extirpated from the type locality due to a series of impoundments along the Ouachita River. Both species are typically small, length usually not exceeding 50–55 mm. Shell of *O. jacksoniana* is moderately thick, laterally inflated, and shape is oval to subtriangular (Williams et al. 82). In contrast, *V. arkansasensis* shell is relatively thin, laterally compressed, and shape is subtriangular (Robison and Allen 60). Both species exhibit sexual dimorphism with female *O. jacksoniana* more inflated and broader in the posterior margin and female *V. arkansasensis* with an indentation and sulcus emanating from the posterior ventral margin. As both species are distributed in the southeastern United States and have similar shell characteristics, they can be difficult to distinguish in the field, especially in small specimens.

The objectives of this study were to investigate (1) phylogenetic structure and evolutionary divergence of *O. jacksoniana* and *V. arkansasensis* using two mitochondrial DNA (mtDNA) genes; and (2) morphological similarity between the two species using traditional morphometric analysis. We tested the null hypothesis that phylogenetic relationships between *O. jacksoniana* and *V. arkansasensis* reflect current taxonomic status and the similarity of shell morphology between the species is due to convergence. Furthermore, we investigated correlations between morphological variations and geographic distance along the rivers. As the conservation status of both species is considered special concern (Williams et al. 81; Harris et al. 33), and they co-occur in certain river drainages, our aim was to confirm their taxonomic status and provide shell morphological characteristics for accurate identification that is integral to population assessment for conservation management.

## Methods

### Sampling

**Figure 2:**
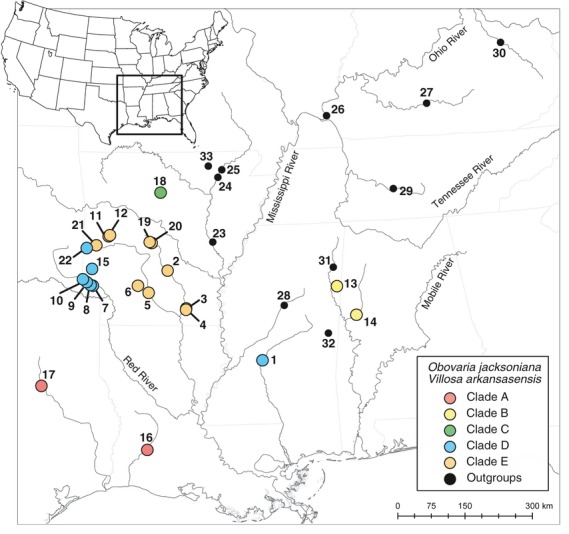
Collection sites in the southern United States. Colors correspond to phylogenetic clades (Fig. 3). Numbers correspond to locality information (see Table S1).

We used 116 individuals of *O. jacksoniana* representing 12 streams from seven major drainages and 19 individuals of *V. arkansasensis* representing three streams from two major drainages with initial species assignments based on shell morphology (Fig. 2; see Table S1). Specimens were either collected specifically for this study or obtained from museum collections. We obtained *O. jacksoniana* paralectotypes (University of Michigan Museum of Zoology, UMMZ 107523) and *V. arkansasensis* (near) topotypic specimens (Arkansas State University Museum of Zoology, ASUMZ 4579-4583) for inclusion. All putative *Obovaria* species (*O. jacksoniana*,* O*. *olivaria* [Rafinesque 1820], *O*. *retusa* [LaMarck 1819], *O*. *subrotunda* [Rafinesque 1820], and *O*. *unicolor* [Lea 1845]), except *O*. *haddletoni* (Athearn 1964), and four putative *Villosa* species (*V*. *fabalis* [Lea 1831], *V*. *iris* [Lea 1829], *V*. *vanuxemensis* [Lea 1838], and *V*. *villosa* [Wright 1898]) were included in the phylogenetic analysis. “*Obovaria” rotulata* (Wright 1899) was also included in this analysis, and it has been placed in both *Fusconaia* (Williams et al. 82) and *Obovaria* (Graf and Cummings 28), but more recently it has been assigned to the new genus *Reginaia* (Campbell and Lydeard 11,b; Haag 31). It should also be noted that recent phylogenetic analyses of *Villosa* have shown paraphyly and taxonomic revision is needed (Zanatta and Murphy 85; Kuehnl 41). Outgroups in the phylogenetic analysis included representatives from seven tribes in the North American Unionidae (see Table S2).

Newly collected specimens were separated into soft tissues and shells with soft tissues preserved in absolute ethanol in −20°C freezer and shells scrubbed inside and out to remove excess material. In most instances, we used the same specimens for both molecular phylogenetic and morphometric analyses.

### DNA Extraction and sequencing

We extracted whole genomic DNA from ethanol-preserved mantle tissue using standard CTAB/chloroform extraction followed by ethanol precipitation (Saghai-Maroof et al. 63). We obtained dried muscle tissues from adductor scars of paralectotype *O. jacksoniana* specimens. Dried tissues were soaked in CTAB buffer and incubated at 55°C overnight prior to extraction. We amplified two mtDNA genes, cytochrome *c* oxidase subunit I (COI), and NADH dehydrogenase subunit 1 (ND1), following the specifications included with *Taq* DNA polymerase (QIAGEN, Inc., Valencia, CA) in a 20-μL reaction. We used the COI and ND1 primers, reaction conditions, reagent concentrations, and thermal cycles described in Campbell et al. (13). We cleaned PCR products using a QIAquick gel extraction kit (QIAGEN, Inc.) per 26 μL of PCR product. We used the same primers as PCR for sequencing reactions. Sequencing products were analyzed on a CEQ™ 8000 automated sequencer (Beckman Coulter, Inc., Brea, CA). DNA sequences were assembled, edited, and aligned by eye using the program DNADynamo (Blue Tractor Software, Ltd., Llanfairfechan, U. K.), and an open reading frame for these genes was verified. Ambiguous sequences of both the 3′- and 5′-ends were trimmed.

### Phylogenetic analyses

We conducted the following statistical tests prior to phylogenetic analyses in order to qualify the genetic data. The test for nucleotide saturation was based on Xia et al. (84) implemented in DAMBE v.5.2.18 (Xia and Xie 83). Nucleotide saturation is a substantial problem in loss of phylogenetic information contained in sequence data resulting in misleading phylogenetic relationships. Substitution saturations were determined for each gene and each codon of genes.

We analyzed phylogenetic relationships using maximum likelihood analysis (ML) and Bayesian inference analysis (BI). MtDNA sequence data from COI and ND1 gene portions were analyzed as a concatenated dataset of unique haplotypes only using TCS v.1.21 (Clement et al. 15). For estimating best-fit models of nucleotide substitution, we used Kakusan4 (Tanabe 73). Kakusan4 computes substitution models for multiple loci and each codon partition and estimates likelihoods of substitution models and automatically generates TreeFinder and NEXUS files to directly analyze data using each phylogenetic program. Based on the Akaike information criteria (AIC), best-fit models for a concatenated dataset were GTR+Γ for all codon positions, except HKY85 for the second codon of COI. These best-fit models were used in both ML and BI. ML was performed with TreeFinder (Jobb et al. 39; Jobb 38) using the default settings and the files generated by Kakusan4. Support values were generated by pseudo-bootstrapping with 1000 replicates using the expected-likelihood weights with local rearrangements of tree topology (LR-ELW) implemented in TreeFinder. The LR-ELW edge support can be directly interpreted as confidence in the configuration of branches adjacent to a particular edge. BI was performed with MrBayes v3.1.2 (Huelsenbeck and Ronquist 37) by Markov Chain Monte Carlo. Two simultaneous Markov chains (each chain contains three heated chains and one cold chain) were run for four million generations with trees sampled every 1000th generation yielding 4001 trees for each chain in the initial samples. We assessed burn-in by plotting the log likelihood scores for each sampling point using Tracer v1.5 (Rambaut and Drummond 58), and we considered the Markov chains stationary when like-lihood values reached a plateau. Therefore, we discarded the first 1001 trees (25%) as burn-in for each run, and the remaining 3000 trees were calculated using the 50% majority rule consensus trees. BI trees were compared with the ML tree, and the most credible inferences of relationship were confined to nodes where both the LR-ELW bootstrap support values were >70 and the Bayesian posterior probabilities were >0.95.

### Evolutionary divergence

We estimated genetic diversity within *Obovaria* species and clades of the *O. jacksoniana*/*V. arkansasensis* complex. We calculated the number of substitutions per site by averaging overall sequence pairs within group. We also estimated evolutionary divergence among *Obovaria* species and clades within the *O. jacksoniana*/*V. arkansasensis* complex using the concatenated dataset and the maximum composite likelihood method in MEGA v5.05 (Tamura et al. 72).

### Morphometric analysis

We measured three shell characters on only the *O. jacksoniana/V. arkansasensis* complex from all of the locations. We measured each shell to the nearest 0.05 mm for maximum length (anterior to posterior), height (dorsal to ventral), and width (right to left valve) using dial or digital calipers. To standardize the variables for size, we calculated the height/length, width/length, and width/height ratios for all specimens. The ratio data were normalized using arcsine transformation, and normal distribution was verified for each ratio using Shapiro–Wilk test (Sokal and Rohlf 67). This method has been used to distinguish morphologies of cryptic species in freshwater mussels (Gangloff et al. 24) and to evaluate morphological variations within species (Hornbach et al. 35). We examined morphological variation within and among species through principal component analysis (PCA) and canonical variates analysis (CVA). PCA simplifies descriptions of variation among individuals, while CVA simplifies descriptions of variation between groups (Zelditch et al. 87). In PCA, no a priori assumptions are needed to group individuals. Meanwhile, an a priori assumption of group membership is required for CVA, as it determines the set of axes that best discriminate between groups. We performed a CVA on a data set with groups assigned by phylogenetic clades. Additionally, we utilized Hotelling's tests for pairwise comparisons between groups assigned by species and by phylogenetic clades, and we conducted discriminant function analysis (DFA) for each pair of groups to determine how frequently PC scores correctly distinguished between species. All statistical analyses were performed using PAST software package (Hammer et al. 32).

### Test of correlations

We tested correlations of geographic distance, morphological distance, and genetic distance using Mantel tests (Mantel 45) to examine the effect of landscape features on genetic and morphological structure in *O. jacksoniana* and *V. arkansasensis*. For geographic distance, we measured total distance between populations along the rivers (river distance) using ArcGIS v9.3 (ESRI, Inc., Redlands, CA) because direct geographic distance is often not the best indicator of isolation by distance in aquatic organisms (Spear et al. 68; Storfer et al. 69). This is especially true of mussels as they are fully aquatic and incapable of migration over terrestrial habitats. When two populations were not connected by rivers, we took the closest distance through the Gulf of Mexico. For morphological distance, we calculated the Euclidean distance between individuals from PCA plots using R (R Development Core Team 57). We calculated pairwise genetic distances between individuals from the concatenated data set with a maximum composite likelihood model using MEGA v5.05 (Tamura et al. 72). We performed Mantel tests for a combination of three matrices: (1) between river distance and genetic distance (hereafter R × G); (2) between river distance and morphological distance (R × M); and (3) between morphological distance and genetic distance (M × G). In addition, because we had a number of populations represented in the Saline and Little rivers (see Table S1), we performed Mantel tests for each matrix combination on these rivers separately from the overall data set. Mantel tests were performed in the Microsoft Excel add-in program GenAlEx (Peakall and Smouse 56) using 9999 permutations.

## Results

### Phylogenetic analyses

We obtained 645 bp of the COI gene from 135 individuals and 771 bp of the ND1 gene from 125 individuals. We concatenated both genes and obtained a total of 116 unique haplotypes. All DNA sequences were deposited in NCBI GenBank (accession numbers: KF035133–KF035280 for COI and KF035281–KF035420 for ND1). The test for substitution saturation showed no indication of saturation among codons (data not shown). Thus, we used all codon positions in our analyses.

**Figure 3:**
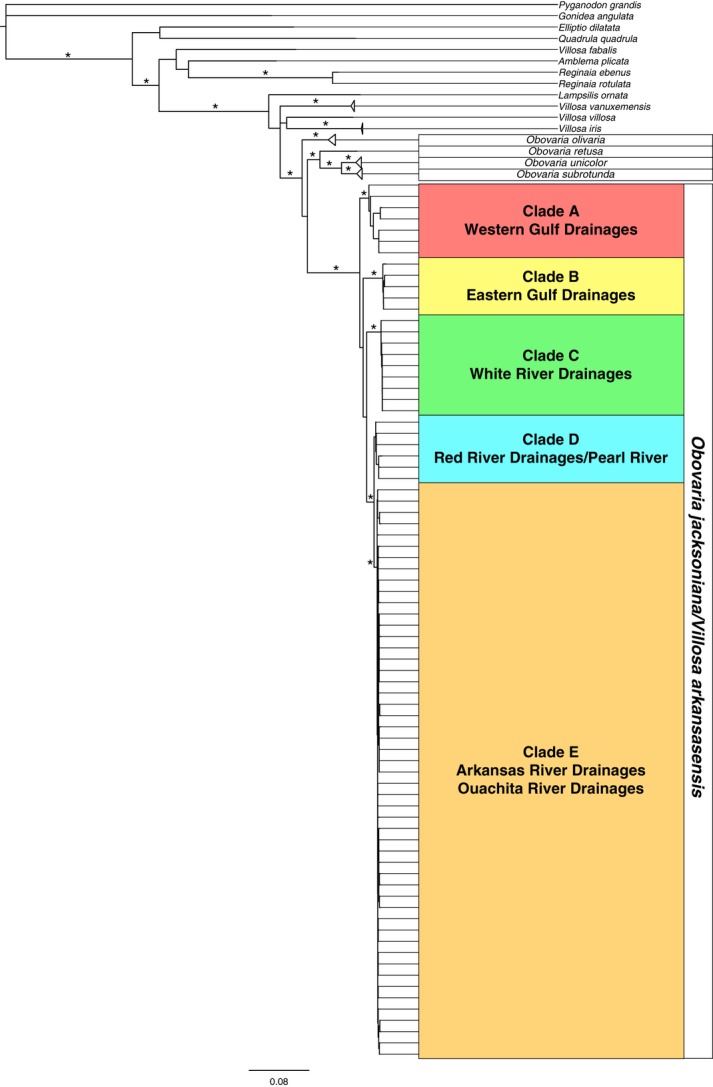
Phylogenetic tree generated by Bayesian and maximum likelihood analyses for concatenated genes. Asterisks indicate branches with bootstrap support values >70 for maximum likelihood analysis and posterior probabilities >0.95 for Bayesian analysis. *Pyganodon grandis* is the outgroup. Colors on *Obovaria jacksoniana/Villosa arkansasensis* clades correspond to Fig. 2.

Phylogenetic analyses showed that *O. jacksoniana* and *V. arkansasensis* formed a single monophyletic clade (Fig. 3). *Obovaria* species (including *V. arkansasensis*) showed a monophyletic relationship with high support values; however, *Villosa* species included in our study were polyphyletic forming four clades. Among *Obovaria, O*. *olivaria* comprised the basal clade in the ML tree followed by a clade comprised of *O*. *retusa, O*. *unicolor*, and *O*. *subrotunda,* and finally a clade of *O. jacksoniana* and *V. arkansasensis*. According to both ML and BI trees, each *Obovaria* species forms a reciprocally monophyletic clade with high support values validating current taxonomic concepts.

Phylogeographic analysis revealed five distinct clades with high support values associated with major drainages for the *O. jacksoniana*/*V. arkansasensis* complex. Individuals from the Western Gulf drainages (e.g., Calcasieu and Neches rivers) comprised the basal clade of the *O. jacksoniana/V. arkansasensis* complex, which continued to individuals from the Mobile River (Eastern Gulf) drainage and from the White River drainage (Fig. 3). Individuals from the Red and Ouachita River drainages were located as sister clades. Individuals from the Fourche La Fave River, a tributary of the Arkansas River, fell into the same clade as the Ouachita populations (Fig. 3 Clade E). Surprisingly, a paralectotype individual from the Pearl River, MS was more closely related to individuals from the Cossatot River and Mountain Fork of the Little River (Red River drainages). No individuals from the same population were located in more than one major drainage clade.

### Evolutionary divergence

We estimated intraspecific genetic diversity over a concatenated data set (Table 1). As the phylogenetic analyses showed five *Obovaria* clades representing recognized species, and five additional clades within the *O. jacksoniana/V. arkansasensis* complex, we estimated intraclade genetic diversity as well. Intraspecific genetic diversity ranged from 0.005 (*O*. *unicolor*) to 0.02 (*O. jacksoniana/V. arkansasensis* complex). Within the *O. jacksoniana/V. arkansasensis* clades, genetic diversity ranged from 0.001 (Clade C) to 0.009 (Clade A). Overall, *O. jacksoniana/V. arkansasensis* had relatively higher genetic diversity when compared to other *Obovaria* species.

**Table 1:** Intraspecific nucleotide diversity and evolutionary divergence estimates (SE estimates in parentheses) over concatenated sequence pairs among genus *Obovaria*

Species	Clade	*Obovaria olivaria*	*Obovaria retusa*	*Obovaria subrotunda*	*Obovaria unicolor*	*Obovaria jacksoniana/Villosa arkansasensis*											
						Overall	Clade A	Clade B	Clade C	Clade D	Clade E
*Obovaria olivaria*		0.007 (0.0014)									
*Obovaria retusa*		0.088 (0.0086)	n.a.								
*Obovaria subrotunda*		0.087 (0.0085)	0.081 (0.0085)	0.006 (0.0014)							
*Obovaria unicolor*		0.084 (0.0078)	0.082 (0.0083)	0.038 (0.0050)	0.005 (0.0009)						
*Obovaria jacksoniana Villosa arkansasensis*	Overall	0.093 (0.0073)	0.109 (0.0092)	0.106 (0.0079)	0.100 (0.0079)	0.020 (0.0020)					
	Clade A	0.086 (0.0098)	0.107 (0.0124)	0.092 (0.0097)	0.091 (0.0101)	n.a.	0.009 (0.0027)				
	Clade B	0.095 (0.0091)	0.118 (0.0115)	0.116 (0.0105)	0.107 (0.0097)	n.a.	0.046 (0.0067)	0.004 (0.0012)			
	Clade C	0.093 (0.0095)	0.107 (0.0109)	0.103 (0.0098)	0.095 (0.0091)	n.a.	0.040 (0.0070)	0.043 (0.0057)	0.001 (0.0005)		
	Clade D	0.087 (0.0089)	0.106 (0.0112)	0.100 (0.0094)	0.094 (0.0087)	n.a.	0.036 (0.0057)	0.041 (0.0056)	0.029 (0.0049)	0.004 (0.0014)	
	Clade E	0.095 (0.0090)	0.109 (0.0109)	0.108 (0.0094)	0.102 (0.0091)	n.a.	0.039 (0.0061)	0.043 (0.0059)	0.032 (0.0050)	0.011 (0.0022)	0.004 (0.0007)

Clades in *Obovaria jacksoniana/Villosa arkansasensis* complex correspond to phylogenetic tree (Fig. 3). n.a., not applicable.

We estimated evolutionary divergence between clades of *Obovaria* (Table 1). Evolutionary divergences ranged from 0.038 to 0.109 with a mean of 0.076. We observed the highest divergence between *O*. *retusa* and *O. jacksoniana/V. arkansasensis* complex (0.109, SE = 0.0092), and the lowest divergence between *O*. *subrotunda* and *O*. *unicolor* (0.038, SE = 0.005). Within the *O. jacksoniana*/*V. arkansasensis* clades, we observed relatively high evolutionary divergences between Clade A and Clade B (0.046, SE = 0.0067), between Clade B and Clade C (0.043, SE = 0.0057), and between Clade B and Clade E (0.043, SE = 0.0059). These evolutionary divergences were higher than between *O*. *subrotunda* and *O*. *unicolor*. We observed small divergences between Clade D and Clade E (0.011, SE = 0.0022).

### Morphometric analyses

**Figure 4:**
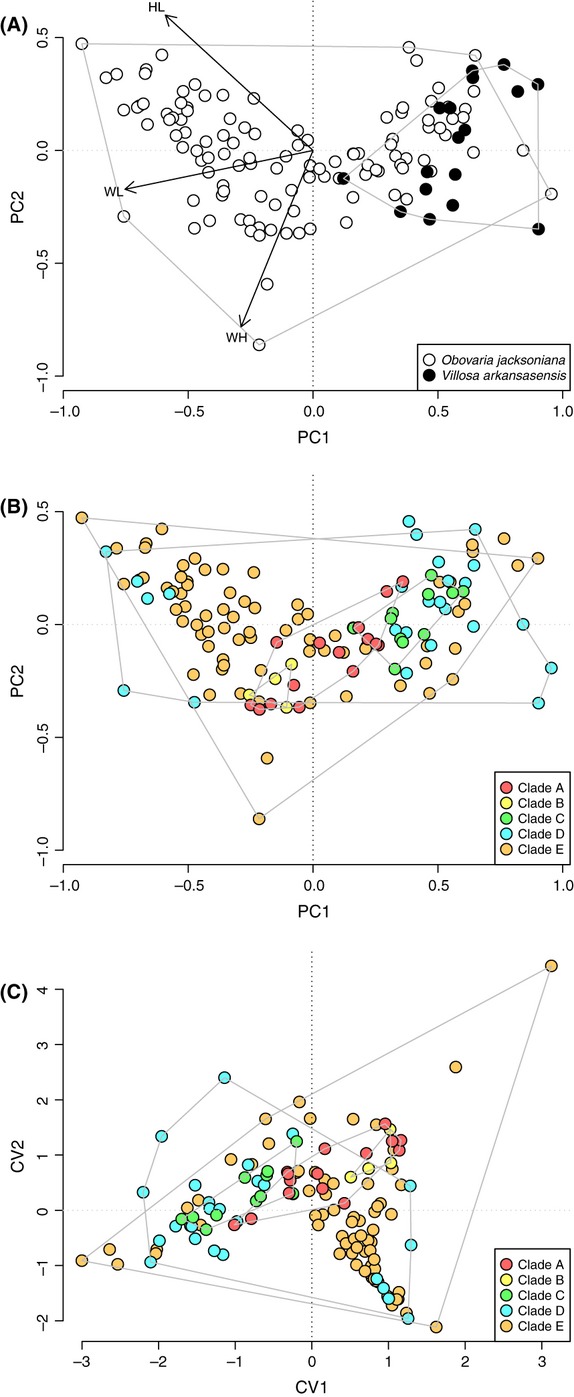
Scatter plots from principal component analysis (PCA; A and B) and canonical variates analysis (CVA; C) of *Obovaria jacksoniana* (*n* = 114) and *Villosa arkansasensis* (*n* = 17). Polygons enclose convex hull of each group assigned. PCA plots were grouped assigned by species (A) and by phylogenetic clades (B). Arrows showed a biplot of variables on PCA (HL, height/length, WL, width/length, and WH, width/height). The PC1 axis described 80.05% and the PC2 axis described 19.94% of total variation. (C) CVA plot was analyzed with groups assigned by phylogenetic clades. The CV1 axis described 68.14% and the CV2 axis described 31.86% of total variation. Colors of phylogenetic clades correspond to Fig. 3.

We analyzed a total of 114 individuals identified morphologically as *O. jacksoniana* and 17 individuals identified morphologically as *V. arkansasensis*. PCA yielded two distinct eigenvalues and described >99% of the total variability between species. The PC1 axis described 80.05% and the PC2 axis described 19.94% of the total variation (Fig. 4A and B). The PCA, with group assigned by species, showed a wide morphological range for *O. jacksoniana* and a relatively small morphological range for *V. arkansasensis* (Fig. 4A) with large overlapping cluster portions that included 29.77% of the total individuals. While PCA showed large cluster portions overlapping between the two species, pairwise comparisons (Hotelling's tests) between *O. jacksoniana* and *V. arkansasensis* showed significantly different morphologies (Bonferroni corrected, *P* < 0.005). The DFA scores between two groups revealed 76.34% of individuals were correctly assigned a group, where 29 individuals of *O. jacksoniana* were assigned to *V. arkansasensis* and two individuals of *V. arkansasensis* was assigned to *O. jacksoniana*.

The PCA, with group assigned by clades, showed wide morphological ranges for Clade D and Clade E, while the rest of the clades showed relatively small morphological ranges overlapping with Clade D and Clade E (Fig. 4B). The CVA, with group assigned by clades, yielded two distinct axes and described 96% of the total variability among clades (Fig. 4C). The CV1 axis described 67.41% and the CV2 axis described 29.58% of the total variation. Similar to PCA results, CVA clusters also showed a wide morphological range of Clade D and Clade E and large overlap among clades. Pairwise comparisons among clades showed no significant difference between Clade A, Clade B, and Clade E (Bonferroni corrected, *P* = 1–0.369, see Table S3), while the rest of the pairwise comparisons was significantly different (Bonferroni corrected, *P* < 0.05).

### Test of correlations

**Figure 5:**
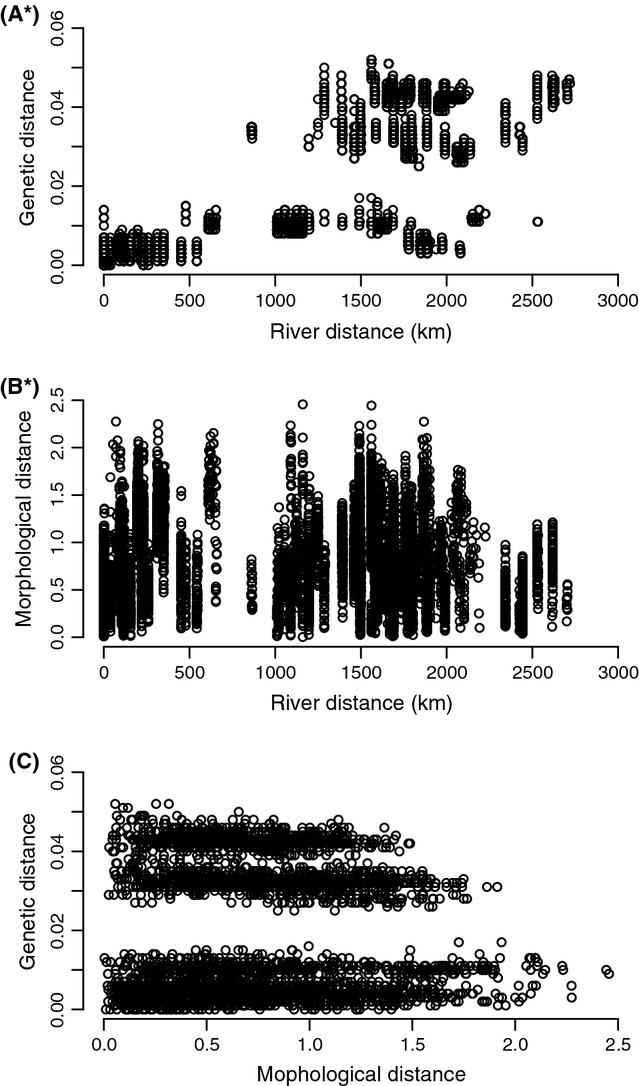
Results of Mantel tests between (A) pairwise river distance among collection sites (km) and pairwise genetic distance, (B) pairwise river distance and pairwise morphological distance, and (C) pairwise morphological distance and pairwise genetic distance. *P < 0.05.

Mantel tests examining correlations of three distance matrices in overall populations indicated significant correlations of R × G and R × M (Fig. 5). R × G showed strong positive correlations indicating strong isolation by distance (Mantel's *r* = 0.837, *P* < 0.001; Fig. 5A). Although R × M correlation was statistically significant, correlation was only slightly positive (Mantel's *r* = 0.079, *P* = 0.017; Fig. 5B). Correlation of M × G was not statistically significant (Mantel's *r* = 0.012, *P* = 0.404; Fig. 5C) but indicated two distinct clusters. The bottom cluster shows that shell morphologies are highly variable within genetically closely related individuals. Meanwhile, the top cluster shows that less related individuals possessed both similar and different shell morphologies.

**Figure 6:**
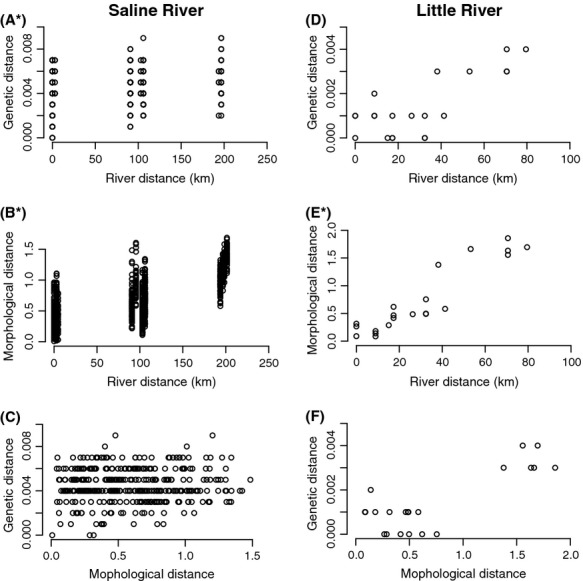
Results of Mantel tests between (A and D) pairwise river distance among collection sites (km) and pairwise genetic distance, (B and E) pairwise river distance and pairwise morphological distance, and (C and F) pairwise morphological distance and pairwise genetic distance for the Saline River and Little River, respectively. *P < 0.05.

Results for the Saline and Little rivers showed similar trends to the overall data set; however, only correlations of R × M for both rivers were statistically significant (Mantel's *r* = 0.761, *P* < 0.001 for Saline River; *r* = 0.929, *P* = 0.002 for Little River; Fig. 6B and E). Correlations of R × G were only significant for the Saline River and marginally significant for the Little River (Mantel's *r* = 0.151, *P* = 0.021 for the Saline River; *r* = 0.772, *P* = 0.057 for the Little River; Fig. 6A and D). This indicates that isolation by distance relationships are weakened at smaller spatial scales. Similar to the overall data set, neither river showed correlations of M × G (Mantel's *r* = 0.038, *P* = 0.337 for Saline River; *r* = 0.783, *P* = 0.143 for Little River; Fig. 6C and F).

## Discussion

### Genetic structure

Phylogenetic analysis did not support the hypothesis that phylogenetic relationships between *O. jacksoniana* and *V. arkansasensis* reflect current taxonomic status; rather, these two species shared a single clade. These two nominal species are morphological variants of the same phylogenetic species. The *O. jacksoniana*/*V. arkansasensis* complex split into five reciprocally monophyletic clades indicating five phylogeographic isolations primarily corresponding to major river drainages. Phylogeographic patterns associated with geographic barriers and/or historical vicariant events are reported in many freshwater organisms such as fish (Mayden 46, 47; Turner and Robison 74; Berendzen et al. 5), crayfish (Crandall and Templeton 17; Fetzner and Crandall 21), and mussels (King et al. 40; Turner et al. 75; Roe et al. 62; Serb 64; Burdick and White 10; Elderkin et al. 20).

In our study, the five *O. jacksoniana*/*V. arkansasensis* clades are distributed in the western Gulf of Mexico (Calcasieu and Neches rivers), eastern Gulf of Mexico (Mobile River), and three major Mississippi River drainage systems (White, Arkansas/Ouachita, and Red rivers), which correspond to the recently established Sabine-Trinity, Mobile Basin, and Interior Highlands mussel provinces (Haag 30), respectively. It is noteworthy that topotypic Pearl River *O. jacksoniana* grouped with the Red River clade. Our results suggest that large rivers such as the Mississippi River and the marine environment of the Gulf of Mexico are substantial barriers to gene flow for this species complex. This is evidenced by a lack of haplotype sharing among river drainages, relatively high levels of evolutionary divergence among drainages [*O. jacksoniana*/*V. arkansasensis* complex (3.4%) compared to *Potamilus* (1.32%) and *Quadrula* (3.65%) (Roe and Lydeard 61; Serb et al. 65)], and significant positive isolation by distance patterns. These patterns of isolation by distance have been observed in other freshwater mussel species; *Actinonaias ligamentina* (LaMarck 1819) (Elderkin et al. 20), *Elliptio dilatata* (Rafinesque 1820) (Berg et al. 6; Elderkin et al. 20), *Epioblasma torulosa rangiana* (Lea 1838) (Zanatta and Murphy 86), and *Unio pictorum* (Linnaeus 1758) (Zieritz et al. 88). Another potential cause of isolation by drainage is the relative immobility of host fishes. While the life history of *O. jacksoniana* is not completely known, specifically with regards to host fish, we make the assumption that *O. jacksoniana* uses the same hosts as *V. arkansasensis*, which are centrarchids (*Ambloplites ariommus* [Viosca 1936] and *Lepomis cyanellus* [Rafinesque 1819]) and benthic percids (*Etheostoma blennioides* [Rafinesque 1819] and *Etheostoma collettei* [Birdsong and Knapp 1969]) (Christian et al. 14). All of these species have small home ranges and poor dispersal abilities (Greenberg 29; Gatz 26).

### Shell morphologies and correlations with landscapes

Various taxa have displayed phenotypic plasticity in response to biotic and abiotic factors (Brönmark and Miner 8; Via et al. 78). Both environmental and landscape factors can influence phenotypic variation (e.g., Hinch et al. 34; Shepherd 66; Minton et al. 48). In freshwater mussels, gradual changes in shell morphologies from upstream to downstream have been observed in several taxa (Ortmann 54; Graf 27; Hornbach et al. 35). Similar to these studies, our results showed clear evidence that spatial and river characteristics strongly influenced morphological variations of these freshwater mussels.

Although Hotelling's test showed significant morphological differences between *O. jacksoniana* and *V. arkansasensis*, 29 individuals (22.8%) were incorrectly assigned to initially identified species, and morphometric analysis through PCA showed significant morphological overlap between the species. The PCA biplot showed that PC1 is mainly weighted by a proportion of lateral inflation and shell length, and some *O. jacksoniana* individuals exhibit less lateral inflation, similar to *V. arkansasensis* shell morphologies. Correlations of R × M at local scales (individual rivers) showed that similarity of shell morphologies gradually declined with increasing river distance between sites; however, this relationship was only slightly positive at the regional scale where similarity of shell morphologies did not decline with increasing distance between rivers. Additionally, we did not observe significant correlations of M × G in either local or regional scales indicating that genetic characters were not the primary control of shell morphological variation, and genetically similar individuals can exhibit either the *O. jacksoniana* or *V. arkansasensis* morphotype. Thus, for this species complex, it is likely that shell morphology is primarily determined by environmental factors, such as river position. Given the evidence, it appears *V. arkansasensis* is the headwater morphotype and *O. jacksoniana* is the larger river morphotype in the *O. jacksoniana/V. arkansasensis* complex.

### Conservation implications

Based on our findings, *V. arkansasensis* and *O. jacksoniana* are synonomous and most closely related to a clade composed of *O*. *retusa*,* O*. *subrotunda*, and *O*. *unicolor*. Therefore, *V. arkansasensis* and *O. jacksoniana* should be recognized as *Obovaria arkansasensis* (Lea 42) n. comb. As *O. jacksoniana* and *V. arkansasensis* are thought to occupy different longitudinal reaches along the stream continuum, synonomy will expand the overall distribution of the taxon within a specific river drainage from headwaters to large river habitats. However, we must be cautious in reassessing the conservation status of *O*.* arkansasensis*. The mtDNA phylogeny showed five reciprocally monophyletic clades and high levels of evolutionary divergence among clades indicating in-progress speciation and/or cryptic species within the *O. jacksoniana/V. arkansasensis* complex. These lineages are evolutionarily significant and require separate conservation management. We should note, however, that the mtDNA phylogeny represents only part of species’ evolutionary history. Therefore, further study using additional specimens from throughout the geographic range and additional genetic markers such as the nuclear DNA genes and microsatellite loci is required to elucidate genetic divergences and to refine species limits. Finally, biological and ecological assessments, such as host fish and habitat specificities among the *O. jacksoniana/V. arkansasensis* complex, are highly recommended, and we suggest host fish species investigations for the *O. jacksoniana/V. arkansasensis* complex from multiple drainages to further assess ecological variance of the species complex.

Recent molecular phylogenetic analyses elucidated cryptic diversity within freshwater mussels (King et al. 40; Gangloff et al. 24). However, discovery of potential synonomous species possessing different phenotypes due to (eco)phenotypic plasticity is rare compared to other taxa. For example, a recent study of deep-sea macroinvertebrates revealed synonomous species in tubeworms and shrimps previously assigned to different species or genera due to developmental plasticity (Vrijenhoek 79). We believe there may be other examples in freshwater mussels, which have yet to be discovered.

## Conclusions

Although shell morphology is often used as the key characteristic for identification and taxonomy of freshwater mussel species, morphologies can be extremely variable in different habitats. Our study shows evidence of ecophenotypic plasticity in freshwater mussel shell morphologies within the same drainages and highlights the difficulty of shell morphology-based identification. Our study was unique in that genetic information provided evidence for synonomy of species, while morphometric data supported two distinct taxa. Based on our study, we conclude that *O. jacksoniana* and *V. arkansasensis* are different morphotypes in the same species complex due to ecophenotypic plasticity and should be synonomized under the nomen *Obovaria arkansasensis* (Lea 42) n. comb. However, we recommend this species retain the conservation status of special concern due to its relative rarity over a large portion of its overall range. Moreover, our study suggests that correlations among distance matrices are of great utility to investigate not only patterns of genetic isolation by distance but also phenotypic plasticity within the species. Genetic isolation by distance has been frequently used to examine dispersal abilities, population fragmentation, and gene flow among populations for other freshwater mussel species (Elderkin et al. 19, 20; Zieritz et al. 88) and other aquatic organisms (Costello et al. 16; Finn et al. 22; Alp et al. 3). However, combinations between genetic and morphological distances have been rarely used to investigate phenotypic plasticity in freshwater mussels (Zieritz et al. 88). Our study revealed cryptic diversity within the *O. jacksoniana*/*V. arkansasensis* complex that has prompted additional investigations of genetic diversity, geographic distribution, and relative abundance to clearly define the taxonomic status of these clades. We believe that the results of this study encourage researchers and conservation agencies to use combinations of genetic, biological, and morphological information for developing conservation strategies of these highly imperiled animals.

## Supporting Information

**Table S1.** List of taxa and specimens utilized in this study. Numbers on map correspond to Fig. 2. **Table S2.** List of published sequences with NCBI accession numbers and references utilized in this study. **Table S3.** Bonferroni corrected *P*-values of the Hotelling's pairwise comparison. Clades in *Obovaria jacksoniana/Villosa arkansasensis* complex correspond to phylogenetic tree (Fig. 3).
