# Supplementary material for: Phylogenetic and morphometric analyses reveal ecophenotypic plasticity in freshwater mussels Obovaria jacksoniana and Villosa arkansasensis (Bivalvia: Unionidae)
Source: Ecol Evol. 2013 Jul 3;3(8):2670–83. doi: 10.1002/ece3.649 (PMC3930048; doi:10.1002/ece3.649)
Supplement: Supplementary file 1 — Table S1. List of taxa and specimens utilized in this study. Numbers on map correspond to Fig. 2. Table S2. List of published sequences with NCBI accession numbers and references utilized in this study. Table S3. Bonferroni corrected P-values of the Hotelling's pairwise comparison. Clades in Obovaria jacksoniana/Villosa arkansasensis complex correspond to phylogenetic tree (Fig. 3). [file ece30003-2670-SD1.docx]

Table S1. List of taxa and specimens utilized in this study. Numbers on map correspond to Fig. 2.

| River/County/State | n | Number on Map | Drainage | Latitude | Longitude |
| --- | --- | --- | --- | --- | --- |
| ***Obovaria jacksoniana*** |  |  |  |  |  |
| Pearl River, Hinds Co., MS | 18 | 1 | East Gulf | 32.2819 | -90.1788 |
| Saline River, Dallas/Grant Co., AR | 14 | 2 | Ouachita | 34.1141 | -92.4095 |
| Saline River, Ashley/Bradley Co., AR | 11 | 3 | Ouachita | 33.3509 | -91.9708 |
| Saline River, Ashley/Bradley Co., AR | 10 | 4 | Ouachita | 33.3266 | -91.9758 |
| Ouachita River, Ouachita Co., AR | 17 | 5 | Ouachita | 33.6700 | -92.8697 |
| Little Missouri River, Nevada Co., AR | 6 | 6 | Ouachita | 33.8096 | -93.1341 |
| Little River, Little River/Sevier Co., AR | 1 | 7 | Red | 33.8030 | -94.2136 |
| Little River, Little River/Sevier Co., AR | 3 | 8 | Red | 33.8214 | -94.2711 |
| Little River, Little River/Sevier Co., AR | 1 | 9 | Red | 33.8772 | -94.3677 |
| Little River, Little River/Sevier Co., AR | 1 | 10 | Red | 33.9352 | -94.4580 |
| Fourche La Fave River, Scott Co., AR | 1 | 11 | Arkansas | 34.8037 | -93.8439 |
| Fourche La Fave River, Scott Co., AR | 1 | 12 | Arkansas | 34.8247 | -93.8144 |
| Buttahatchie River, Monroe Co., MS | 2 | 13 | Mobile | 33.7138 | -88.3414 |
| Sipsey River, Pickens/Greene Co., AL | 4 | 14 | Mobile | 33.1217 | -87.9114 |
| Cossatot River, Sevier Co., AR | 1 | 15 | Red | 34.1446 | -94.2392 |
| Calcasieu River, Allen Co., LA | 10 | 16 | West Gulf | 30.5075 | -92.9067 |
| Neches River, Anderson/Cherokee Co., TX | 5 | 17 | West Gulf | 31.7740 | -95.3948 |
| Archey's Fork of the Little Red River, Van Buren Co., AR | 10 | 18 | White | 35.6827 | -92.5761 |
| ***Villosa arkansasensis*** |  |  |  |  |  |
| Alum Fork of the Saline River, Saline Co., AR | 4 | 19 | Ouachita | 34.6728 | -92.7988 |
| Alum Fork of the Saline River, Saline Co., AR | 8 | 20 | Ouachita | 34.6886 | -92.8437 |
| Irons Fork of the Ouachita River, Polk Co., AR | 6 | 21 | Ouachita | 34.6166 | -94.1394 |
| Mountain Fork of the Little River, Polk Co., AR | 1 | 22 | Red | 34.5621 | -94.3750 |
| ***Obovaria olivaria*** |  |  |  |  |  |
| White River, Monroe Co., AR | 13 | 23 | White | 34.6812 | -91.3145 |
| Black River, Lawrence/Randolph Co., AR | 2 | 24 | White | 35.9800 | -91.1625 |
| Black River, Lawrence/Randolph Co., AR | 2 | 25 | White | 36.1312 | -91.0628 |
| Ohio River, Livingston Co., KY | 1 | 26 | Ohio | 37.1433 | -88.4078 |
| ***Obovaria retusa*** |  |  |  |  |  |
| Green River, Hart Co., KY | 1 | 27 | Ohio | 37.2684 | -85.8856 |
| ***Obovaria subrotunda*** |  |  |  |  |  |
| Big Black River, Montgomery Co., MS | 24 | 28 | Mississippi | 33.3749 | -89.6261 |
| Duck River, Maury Co., TN | 5 | 29 | Tennessee | 35.6115 | -86.8539 |
| Licking River, Fleming/Nicholas Co., KY | 1 | 30 | Ohio | 38.3506 | -83.9008 |
| ***Obovaria unicolor*** |  |  |  |  |  |
| East Fork of the Tombigbee River, Itawamba Co., MS | 1 | 31 | Mobile | 34.1020 | -88.4168 |
| Sipsey River, Pickens/Greene Co., AL | 9 | 14 | Mobile | 33.1217 | -87.9114 |
| Sucarnoochee River, Kemper Co., MS | 1 | 32 | Mobile | 32.7828 | -88.6024 |
| ***Elliptio dilatata*** |  |  |  |  |  |
| South Fork of the Spring River, Fulton Co., AR | 1 | 33 | White | 36.2086 | -91.3949 |
| ***Reginaia ebenus*** |  |  |  |  |  |
| White River, Monroe Co., AR | 1 | 23 | White | 34.6812 | -91.3145 |

Table S2. List of published sequences with NCBI accession numbers and references utilized in this study.

| Species | COI | ND1 | References |
| --- | --- | --- | --- |
| *Amblema plicata* | U56841 | AY158796 | Hoeh et al. (1998); Serb et al. (2003) |
| *Gonidea angulata* | DQ272373 | AY655099 | Gustafson & Iwamoto (2005); Campbell et al. (2005) |
| *Lampsilis ornata* | NC_005335 | NC_005335 | Serb & Lydeard (2003) |
| *Pyganodon grandis* | NC_013661 | NC_013661 | Breton et al. (2009) |
| *Quadrula quadrula* | NC_013658 | NC_013658 | Breton et al. (2009) |
| *Reginaia* *rotulata* | AF232814 | AY158799 | Lydeard et al. (2000); Serb et al. (2003) |
| *Villosa fabalis* | DQ220726 | DQ220723 | Zanatta & Murphy (2006) |
| *Villosa iris* | AF156523 | DQ445176 | Graf & O'Foighil (2000); Buhay unpublished |
| *Villosa iris* | AF156524 | DQ445177 | Graf & O'Foighil (2000); Buhay unpublished |
| *Villosa vanuxemensis* | AF156525 | DQ445209 | Graf & O'Foighil (2000); Buhay unpublished |
| *Villosa vanuxemensis* | AF156526 | DQ445210 | Graf & O'Foighil (2000); Buhay unpublished |
| *Villosa villosa* | AF385109 | AY094387 | Roe et al. (2001); Buhay et al. (2002) |

Literature cited

Breton, S., H.D. Beaupré, D.T. Stewart, H. Piontkivska, M. Karmakar, A.E. Bogan, P.U. Blier & W.R. Hoeh (2009) Comparative mitochondrial genomics of freshwater mussels (Bivalvia: Unionoida) with doubly uniparental inheritance of mtDNA: gender-specific open reading frames and putative origins of replication. *Genetics*, **183**, 1575-89.

Buhay, J.E., J.M. Serb, C.R. Dean, Q. Parham & C. Lydeard (2002) Conservation genetics of two endangered unionid bivalve species, *Epioblasma florentina walkeri* and *E. capsaeformis* (Unionidae: Lampsilini). *Journal of Molluscan Studies*, **68**, 385-391.

Campbell, D.C., J.M. Serb, J.E. Buhay, K.J. Roe, R.L. Minton & C. Lydeard (2005) Phylogeny of North American amblemines (Bivalvia: Unionoida): prodigious polyphyly prove pervasive across genera. *Invertebrate Biology*, **124**, 131-164.

Graf, D.L. & D. Ó Foighil (2000) The evolution of brooding characters among the freshwater pearly mussels (Bivalvia: Unionoidea) of North America. *Journal of Molluscan Studies*, **66**, 157-170.

Gustafson, R.G. & E.M. Iwamoto (2005) A DNA-based identification key to Pacific northwest freshwater mussel glochidia: importance to salmonid and mussel conservation. *Northwest Science*, **79**, 233-245.

Hoeh, W.R., M.B. Black, R. Gustafson, A.E. Bogan, R.A. Lutz & R.C. Vrijenhoek (1998) Testing alternative hypotheses of Neotrigonia (Bivalvia: Trigonioida) phylogenetic relationships using cytochrome C oxidase subunit I DNA sequences. *Malacologia*, **40**, 267-278.

Lydeard, C., R.L. Minton & J.D. Williams (2000) Prodigious polyphyly in imperiled freshwater pearly-mussels (Bivalvia: Unionidae): a phylogenetic test of species and generic designations. The Evolutionary Biology of the Bivalvia (ed. by E.M. Harper, J.D. Taylor and J.A. Crame), pp. 145-158. Geological Society, London.

Roe, K.J., P.D. Hartfield & C. Lydeard (2001) Phylogeographic analysis of the threatened and endangered superconglutinate-producing mussels of the genus *Lampsilis* (Bivalvia: Unionidae). *Molecular Ecology*, **10**, 2225-2234.

Serb, J.M. & C. Lydeard (2003) Complete mtDNA sequence of the North American freshwater mussel, *Lampsilis ornata* (Unionidae): an examination of the evolution and phylogenetic utility of mitochondrial genome organization in Bivalvia (Mollusca). *Molecular Biology and Evolution*, **20**, 1854-1866.

Serb, J.M., J.E. Buhay & C. Lydeard (2003) Molecular systematics of the North American freshwater bivalve genus *Quadrula* (Unionidae: Ambleminae) based on mitochondrial ND1 sequences. *Molecular Phylogenetics and Evolution*, **28**, 1-11.

Zanatta, D.T. & R.W. Murphy (2006) Evolution of active host-attraction strategies in the freshwater mussel tribe Lampsilini (Bivalvia: Unionidae). *Molecular Phylogenetics and Evolution*, **41**, 195-208.

Table S3. Bonferroni corrected p-values of the Hotelling’s pairwise comparison. Clades in *Obovaria jacksoniana/Villosa arkansasensis* complex correspond to phylogenetic tree (Fig. 3).

|  | Clade A | Clade B | Clade C | Clade D | Clade E |
| --- | --- | --- | --- | --- | --- |
| Clade A | - |  |  |  |  |
| Clade B | 1.000 | - |  |  |  |
| Clade C | 0.003 | <0.001 | - |  |  |
| Clade D | 0.001 | 0.026 | 1.000 | - |  |
| Clade E | 0.369 | 1.000 | 0.014 | 0.010 | - |
